# The midterm effect of exercise capacity and quality of life in adult patients who underwent hybrid transthoracic device closure of ventricular septal defects

**DOI:** 10.1186/s12872-021-02315-9

**Published:** 2021-10-22

**Authors:** Qiang Chen, Rong Yang, Yu-Qing Lei, Kai-Peng Sun, Hua Cao

**Affiliations:** 1 Department of Cardiac Surgery, Fujian Branch of Shanghai Children’s Medical Center, Fuzhou, China; 2 Fujian Children’s Hospital, Fuzhou, China; 3 Fujian Maternity and Child Health Hospital, Affiliated Hospital of Fujian Medical University, Fuzhou, China; 4 Fujian Key Laboratory of Women and Children’s Critical Diseases Research, Fujian Maternity and Child Health Hospital, Fuzhou, China; 5 Department of Cardiovascular Surgery, Union Hospital, Fujian Medical University, Fuzhou, China; 6 Reproductive Medicine Center, Fujian Maternity and Child Health Hospital, Affiliated Hospital of Fujian Medical University, Fuzhou, China

**Keywords:** QoL, Adult, VSD, Intervention

## Abstract

**Objective:**

To investigate the midterm effect of exercise capacity and quality of life (QoL) of adult patients who underwent transthoracic device closure of ventricular septal defects (VSDs) and explore the gap in the quality of life and cardiopulmonary function between those patients and healthy people.

**Methods:**

From January 2010 to January 2015, 58 adult patients who underwent transthoracic device closure of VSD and 60 healthy people matched for age and sex were selected and analyzed. Echocardiography and exercise capacity tests were performed, and the MOS 36-item short-form health survey (SF-36) was used to investigate the changes in QoL.

**Results:**

Fifty-five patients completed the study. At the 1-year and 5-year follow-ups, the patients’ left ventricular end-systolic and end-diastolic diameters were smaller than those preoperatively, but the difference was not statistically significant. In the QoL survey, the patients’ scores after treatment showed a trend of improvement, and the improvement effect was not transient. After VSD closure, the difference in QoL between the patients and the control group was significantly reduced. However, in the exercise capacity test, the patients’ results were still worse than those of the controls.

**Conclusion:**

Transthoracic device closure of VSDs is significant in improving adult patients’ QoL at the midterm follow-up, reflected in their physical and psychological fields. However, they are still unable to achieve normal levels of peak exercise ability. Therefore, further exploration and interventions are worth considering.

## Introduction

Ventricular septal defects (VSDs) are a common form of congenital heart disease (CHD), most of which are diagnosed and treated in childhood [1]. However, some patients with restrictive VSD fail to be diagnosed in the early stage due to inconspicuous clinical symptoms or insufficient levels of regional diagnosis and treatment, which results in the formation of a cohort of adult patients, and the diagnosis is not made until obvious symptoms occur and periodic physical examination is performed. It is generally believed that surgical repair has an excellent therapeutic effect and can also provide long-term benefits for the patients [2].

With the development of transesophageal echocardiography and the maturity of device techniques, transthoracic device closure has been recognized as another safe and effective treatment for VSD in recent years [3, 4]. Most of the existing studies have focused on the disease progression, treatment effect, and prognosis of children [5, 6]. However, there is still a lack of studies on the prognosis of adult patients, and such treatment affects the exercise capacity and quality of life (QoL) of adult patients. The purpose of this study was: (1) to investigate the midterm effect of transthoracic device closure of VSD on exercise capacity and QoL of adult patients. (2) to explore the gap in the quality of life and cardiopulmonary function between those patients and healthy people.

## Materials and methods

### Patients

This is a retrospective study. Our data were accessed from the patient medical record system. Fifty-eight adult patients who underwent transthoracic device closure of VSDs were enrolled between January 2010 and January 2015. The indication for transthoracic VSD device closure were as follows: (1) a significant left to right shunt and pulmonary-to-systemic blood flow ratio greater than 1.5. (2) the diameter of the VSD was less than 10 mm, and the distance from the aortic valve to the upper rim of the VSD was greater than 2 mm. (3) echocardiography and chest X-ray indicated left heart enlargement and volume overload. (4) complicated with mild-to-moderate pulmonary hypertension. (5) the presence of clinical symptoms, such as shortness of breath and chest tightness. (6) patients refused percutaneous occlusion due to radiation exposure. (7) patients refused surgical repair under cardiopulmonary bypass. The exclusion for VSD device closure were as follows: (1) patients with a VSD accompanied by aortic regurgitation. (2) severe pulmonary hypertension and right-to-left shunt or irreversible pulmonary vascular disease. (3) contraindications to antiplatelet or anticoagulant therapy. (4) active bacterial infection or endocarditis.

The inclusion criteria for this study were as follows: (1) the patient successfully underwent transthoracic closure of the VSD. (2) postoperative echocardiography confirmed that there was no residual shunt. (3) the patient’s physical movement ability was normal, and there was no other systemic disease or deformity affecting their movement function. The exclusion criteria or this study were as follows: (1) patients with cognitive impairment or communication disorders. (2) the patient had other congenital heart malformations or acquired heart disease. (3) the patient had pulmonary disease. (4) the patient was pregnant or breastfeeding. Simultaneously, a group of healthy people with matching age and sex recruited from the outpatient clinic or physical examination center was recruited as the control group. Informed consent was obtained from all participants.

### Transthoracic device closure of VSD

The occluder used in this study was made by Shan Dong Visee Medical Apparatus Co., Ltd., Shanghai Shape Memory Alloy Co., Ltd. and Shenzhen Lifetech Scientific Co., Ltd. of China, which contained symmetric and asymmetric types.

The procedure was performed under general anesthesia. A lower partial median incision was used, and the sternum was partially incised. The pericardium was opened and suspended to expose the right ventricle. A puncture was made in the right ventricle, and the guidewire was inserted through the puncture site. With the guidance of transesophageal echocardiography, the guidewire was advanced through the VSD into the left ventricle. The sheath was inserted along the guidewire to establish the transport track. Then, the occluder was inserted along the sheath and released into the two sides of the ventricular septum to close the VSD [7, 8].

### Echocardiographic measurement

Echocardiography was performed within 7 days before surgery and again at 1 year and 5 years after surgery. The following echocardiographic parameters were recorded: (1) The end-systolic length and width of the right atrium. (2) The end-diastolic length and width of the right ventricle. (3) The end-systolic left atrium volume. (4) The end-systolic and end-diastolic left ventricular dimensions. (5) The left ventricular ejection fraction was calculated. (5) The diameter of the VSD, systolic pulmonary pressure, and Qp/Qs.

### Cardiopulmonary exercise test

A cardiopulmonary exercise test was performed 1 and 5 years after surgery. The test was conducted by a trained professional and was based on existing guidelines [12]. The data were collected using the Jaeger Master Screen®CPX system. Participants underwent a standardized measurement of height and weight. The test used an upright bicycle dynamometer. Before the test, the participants were asked to choose their workload based on their weight, habits, and sex. During the test period, all participants adopted a progressive test scheme, which increased the range from 2 W/6 S to 4 W/8 S, and the exercise time was limited to 8 to 12 min. To maintain a stable state, the pedal speed was kept at 60–70 rpm as far as possible, and the participants were required not to stand, speak or let go of the handlebars. The participants were encouraged to keep pedaling until they were exhausted.

Before each test, the participants were asked to perform a pulmonary function test using the same instrument. The participants’ heart rate, blood oxygen saturation, and 12-lead electrocardiogram were continuously monitored during the cardiopulmonary exercise test. Their blood pressure was measured every two minutes, and their gas exchange parameters were collected during each exhalation, expressed as minute values, with an average interval of 15 s. If the participant’s heart rate or oxygen uptake rate remained stable during the test, the data were considered reliable. A physician was present during each test to avoid adverse events.

### Quality of life assessment tool

The QoL survey was conducted using the MOS 36-item short-form health survey (SF-36), aimed at the physiological and psychosocial fields [9–11]. The physiological areas included four dimensions: physical functioning (PF), role physical (RP), bodily pain (BP), and general health (GH). The field of social psychology encompasses four dimensions: vitality (VT), social functioning (SF), emotional role (RE), and mental health (MH). The study team was comprised of a cardiac surgeon, two cardiac specialist nurses, and a volunteer who was proficient in the local language. The data were collected 7 days before the operation and 1 year and 5 years after the operation.

### Statistical analysis

The sample size was calculated with PASS 22.0. The alpha value was set at 0.05 with a power of 0.90. Combined with the effect index, we calculated that the minimum sample size was 50 patients. Considering a 15% drop rate, we included 58 samples for the study group. The control group was set as 60 samples.

SPSS 22.0 was used for the statistical analysis of the data. Continuous data are expressed as the mean ± standard deviation. *P* < 0.05 was considered statistically significant. The normal analysis of echocardiographic data, cardiopulmonary exercise test data, and QoL data of the patients before and after treatment showed that the distribution of these data was not consistent with the normal distribution. Wilcoxon analysis was used to compare the preoperative and postoperative echocardiographic data. The comparison between the preoperative and postoperative quality of life of the patients was performed using the generalized linear mixed model. However, Mann–Whitney U analysis was used in the comparison between the patient group and the control group.

## Results

In our study, 55 patients in the study group and 58 healthy participants in the control group eventually completed the study. Of the three patients who did not complete the study, two patients discontinued the study due to pregnancy, and one discontinued due to lack of time. In contrast, two healthy participants in the control group could not complete the study due to relocation. In the patient group, the diameter of the VSD was 7.0 ± 1.3 mm, the systolic pulmonary artery pressure was 36 ± 7.0 mmHg, and the Qp/Qs was 1.7 ± 0.5. The demographic data and clinical characteristics of the two groups are recorded in Table 1.

**Table 1:** Demographic and clinical characteristics of the participants

Item	Patient group	Control group	*P*-value
Age (years)	33.8 ± 9.3	32.9 ± 9.2	0.768
Male (%)	30(54.5%)	32(55.2%)	0.832
Weight (kg)	66.5 ± 9.3	63.4 ± 9.0	0.853
Height (cm)	168.5 ± 7.0	169.0 ± 7.5	0.043
Married	48(87.3%)	50(86.2%)	0.513
BMI (kg/m^2^)	22.8 ± 4.5	22.0 ± 5.2	0.378
Qp/Qs	1.7 ± 0.5	–	–
Systolic PAP (mmhg)	39 ± 11.0	–	–
VSD size (mm)	7.0 ± 1.3	–	–
*Type of the VSD*			
Perimembranous VSD	49(89.1%)		
Subarterial VSD	5(9.1%)		
Muscular VSD	1(1.8%)		

VSD: ventricular septal defect; PAP: Pulmonary artery pressure

No severe complications, such as cardiac arrest or aortic valve regurgitation, were reported during the follow-up period. During the one-year and five-year follow-ups, it was found that the end-systolic left ventricular dimensions, the end-diastolic left ventricular dimensions, and the end-systolic left atrial volume were smaller than in the preoperative data, but the difference was not statistically significant. The results also showed no significant change in the left ventricular ejection fraction before and after VSD closure (Table 2).

**Table 2:** Results of echocardiography in both groups

Item	7 days before closure		1 years after closure		5 years after closure		P1	P2
	Patients	Controls	Patients	Controls	Patients	Controls		
End-systolic length of the RA (mm)	44.8 ± 5.7	43.2 ± 6.6	43.6 ± 6.5	43.3 ± 5.1	44.0 ± 5.9	43.5 ± 4.9	0.327	0.435
End-systolic width of the RA (mm)	42.5 ± 5.6	41.3 ± 5.3	41.2 ± 5.0	40.9 ± 4.8	40.8 ± 4.2	41.0 ± 4.5	0.438	0.523
End-diastolic length of the RV (mm)	45.6 ± 5.5	42.5 ± 4.6	43.7 ± 4.0	41.9 ± 5.0	42.1 ± 4.8	41.6 ± 3.9	0.278	0.327
End-diastolic width of the RV (mm)	43.2 ± 3.9	41.3 ± 4.2	40.8 ± 4.5	40.0 ± 3.6	39.1 ± 3.5	40.2 ± 4.3	0.076	0.083
End-systolic LA volume (ml)	40.2 ± 8.7	37.6 ± 7.5*	38.8 ± 9.5	38.2 ± 6.9	37.6 ± 10.0	37.5 ± 9.5	0.082	0.072
End-systolic LV dimensions (mm)	36.1 ± 4.5	33.5 ± 4.8*	34.8 ± 4.1	33.7 ± 5.3	34.6 ± 4.6	34.0 ± 8.9	0.069	0.059
End-diastolic LV dimensions (mm)	54.5 ± 4.8	51.0 ± 5.2	53.8 ± 5.5	50.5 ± 6.0	49.0 ± 6.1	49.8 ± 7.7	0.078	0.064
LV ejection fraction (%)	61.9 ± 6.0	62.0 ± 4.9	60.7 ± 6.8	61.5 ± 6.0	62.5 ± 7.2	63.2 ± 5.5	0.375	0.287
Heart rate (bpm)	75.3 ± 14.1	73.4 ± 12.5	72.6 ± 12.7	74.5 ± 11.8	77.2 ± 15.8	75.6 ± 13.7	0.421	0.531

LV: left ventricle; RV: right ventricle; RA: right atrium; LA: left atrium; P1: P value of 1 year after closure compared with 7 days before closure in patient group; P2: P value of 5 year after closure compared with 7 days before closure in patient group; * representative p < 0.05

**Fig. 1:**
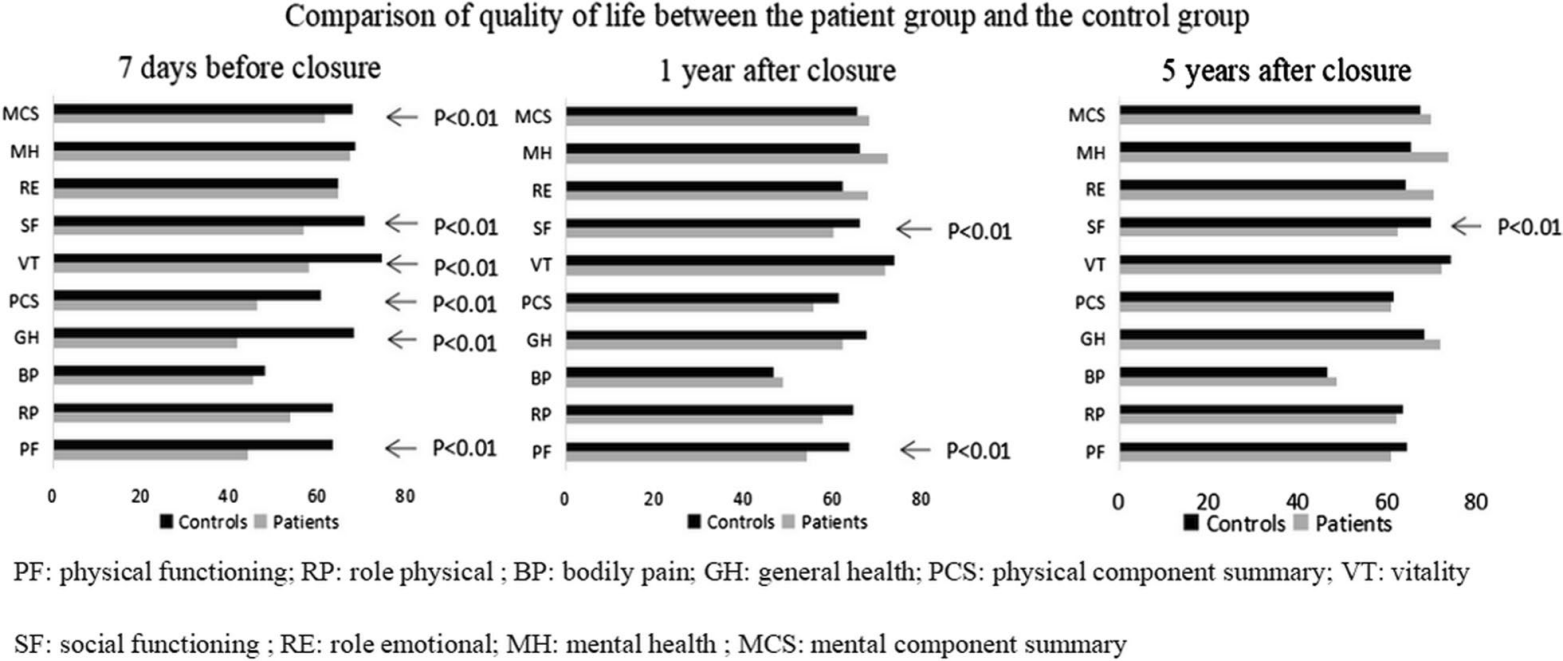
Comparison of quality of life between the patient group and the control group

Table 3 shows the comparison of the QoL of the patients before and after VSD closure. The scores of the patients improved during the one-year and five-year follow-ups, and the improvement in many dimensions was statistically significant. Figure 1 shows a comparison of the quality of life scores between the patient group and the control group. The QoL of the patients lagged behind that of the control group to some extent before the treatment. However, one year after VSD closure, the difference between the patients and the healthy participants gradually decreased, and the difference was statistically significant in the dimensions of “SF’ and “PF”. Five years after VSD treatment, a significant difference between the two groups occurred only in the dimension of “SF”.

**Table 3:** SF-36 scores in patient group and control group

HRQoL Domain	7 days before closure		1 year after closure		5 years after closure		P1	P2	P3
	Patients	Controls	Patients	Controls	Patients	Controls			
Physical functioning	44.15 ± 17.48	63.43 ± 21.54*	54.21 ± 14.24	63.74 ± 16.35*	60.73 ± 13.79	64.28 ± 15.47	< 0.001	< 0.001	< 0.001
Role-physical	53.66 ± 24.88	63.20 ± 24.16	57.62 ± 19.91	64.48 ± 20.75	61.89 ± 15.82	63.57 ± 21.22	0.428	0.074	0.646
Bodily pain	45.27 ± 19.08	48.12 ± 21.75	48.81 ± 17.65	46.68 ± 17.86	48.57 ± 16.18	46.58 ± 15.98	0.267	0.642	0.769
General health	41.65 ± 16.10	68.15 ± 18.90*	62.13 ± 10.42	67.43 ± 14.95	71.73 ± 9.78	68.25 ± 12.54	< 0.001	< 0.001	< 0.001
Physical component summary	46.18 ± 11.30	60.72 ± 11.46*	55.69 ± 8.97	61.37 ± 13.10	60.73 ± 8.98	61.45 ± 8.25	< 0.001	< 0.001	< 0.001
Vitality	57.99 ± 12.62	74.44 ± 16.44*	71.65 ± 12.12	73.86 ± 12.88	72.20 ± 10.97	74.15 ± 12.23	< 0.001	< 0.001	0.278
Social functioning	56.77 ± 15.15	70.42 ± 17.63*	60.06 ± 13.59	66.12 ± 15.28*	62.44 ± 11.96	69.85 ± 14.85*	0.412	0.077	0.362
Role-emotional	64.64 ± 30.26	64.43 ± 20.61	67.89 ± 24.27	62.25 ± 26.12	70.33 ± 24.58	64.13 ± 25.89	0.373	0.156	0.285
Mental health	67.22 ± 16.43	68.34 ± 18.40	72.24 ± 12.13	66.13 ± 15.21	73.56 ± 10.30	65.23 ± 14.28	0.287	0.001	0.065
Mental component summary	61.65 ± 11.53	67.91 ± 8.30*	67.96 ± 9.82	65.45 ± 10.85	69.63 ± 9.35	67.39 ± 8.73	0.096	0.083	0.071

P1: P value of 1 year after closure compared with 7 days before closure in patient group; P2: P value of 5 year after closure compared with 7 days before closure in patient group. P3: P value of 5 year after closure compared with 1 year after closure in patient group; HRQOL: health related quality of life; Date are expressed as Mean ± standard deviation, score ranged from 0 to 100, higher scores indicating better health status, *p* < 0.05 was considered statistically significant. * representative *p* < 0.01

The results of the pulmonary function test are shown in Table 4. The results of the two groups were similar when the measured values were correlated with the standardized predictive values 1 year and 5 years after VSD closure. For the cardiopulmonary exercise test results, the patients performed significantly worse than the healthy controls at the one-year follow-up, with lower maximal load and peak oxygen uptake. For the absolute anaerobic threshold, the oxygen uptake, maximal workload, and heart rate of the patients were lower than those of the control group, and the difference was statistically significant. During the 5-year follow-up, cardiopulmonary exercise capacity was also worse in the patient group than in the healthy controls. However, there was no significant difference in peak heart rate or peak respiratory exchange ratio between the two groups.

**Table 4:** Spirometric outcomes and exercise outcomes of patients and healthy controls

Item	1 years after VSD closure			5 years after VSD closure		
	Patients	Controls	*p*	Patients	Controls	*p*
FVC(%)	112.62 ± 9.43	113.27 ± 10.04	0.789	114.46 ± 10.10	114.64 ± 10.12	0.965
FEV1(%)	92.77 ± 8.92	93.71 ± 9.14	0.640	94.13 ± 9.09	93.19 ± 10.40	0.532
PEF(%)	105.65 ± 9.35	106.88 ± 10.39	0.334	104.80 ± 8.56	104.51 ± 9.19	0.782
FEV1/FVC	82.32 ± 2.58	82.88 ± 6.08	0.531	82.23 ± 3.21	81.44 ± 7.49	0.154
Maximal workload(W)	218.17 ± 33.60	284.94 ± 45.28	< 0.001	223.44 ± 33.73	280.44 ± 45.60	< 0.001
Maximal workload(W/kg)	3.28 ± 0.10	4.49 ± 0.16	< 0.001	3.36 ± 0.10	4.42 ± 0.21	< 0.001
Peak heart rate (beats/min)	179 ± 6.50	182 ± 8.69	0.083	180 ± 8.61	181 ± 10.03	0.289
Minute ventilation (L/kg/minute)	1.50 ± 0.27	1.89 ± 0.36	< 0.001	1.55 ± 0.31	2.02 ± 0.33	< 0.001
Peak oxygen uptake (ml/min)	2496 ± 284	3218 ± 440	< 0.001	2531 ± 288	3299 ± 370	< 0.001
Peak oxygen uptake(ml/kg/min)	37.76 ± 2.35	50.89 ± 2.48	< 0.001	38.30 ± 2.63	52.36 ± 3.45	< 0.001
Peak carbon dioxide excretion(ml/kg/min)	50.62 ± 4.16	69.41 ± 5.29	< 0.001	51.42 ± 5.06	71.90 ± 6.66	< 0.001
Peak respiratory-exchange-ratio	1.34 ± 0.06	1.36 ± 0.07	0.058	1.34 ± 0.008	1.37 ± 0.08	0.078
*Absolute anaerobic threshold*						
Oxygen uptake(ml/kg/min)	22.55 ± 4.33	30.64 ± 6.13	< 0.001	22.21 ± 4.53	32.04 ± 5.67	< 0.001
Maximal workload(W/kg)	1.99 ± 0.20	2.71 ± 0.56	< 0.001	1.91 ± 0.44	2.67 ± 0.67	< 0.001
Heart rate(beats/min)	140 ± 10.98	158 ± 11.34	< 0.001	143 ± 11.93	160 ± 13.38	< 0.001

FVC: forced vital capacity; FEV1: forced expiratory volume in the first second; PEF: peak expiratory flow

In this study, we also compared the postoperative follow-up data of VSD patients with different severity. Some patients with larger VSD performed slightly worse in quality of life and exercise capacity tests than those with smaller VSD, but there was no significant difference.

## Discussion

Although VSD is a congenital disease, it can be considered a chronic disease for some adult patients due to imperfect diagnosis and treatment conditions and insufficient attention. The hemodynamic changes caused by the long-term existence of congenital heart malformations also leads to structural changes in the heart to some extent [13]. The results of this study showed that some patients’ left ventricular size before treatment was at the high limit of the normal value, which was associated with left-to-right shunt changes in cardiac blood flow caused by VSD. After transthoracic device closure, the left ventricular size of the patients showed a trend of reduction.

Unlike children, CHD often affects adult patients beyond the limitations of the disease’s clinical symptoms and physical functions. Westhoff-Black et al.’s research showed that mental symptoms, especially mood and anxiety, were more common in patients with CHD than in the general population [14]. At the same time, the deficiency of interpersonal communication, employment, insurance, and other functions caused by congenital malformations is also an essential factor hindering people’s access to comprehensive health. Researchers have shown that perceived illness, religion, and spirituality are significant predictors of QoL [15]. In a cross-sectional study, 49.2% of patients considered themselves to be religious/spiritual. The results showed that having religious/spiritual beliefs was positively associated with quality of life, life satisfaction, and health behaviors. However, among patients living in more secular countries, religion/spirituality was negatively associated with physical and mental health [16]. Thus, QoL is a diversified concept for evaluating the prognosis of the patient.

Studies on the prognosis of VSD closure indicated that most patients’ clinical status was significantly improved after treatment, and there were also positive changes in their intracardiac structure and cardiac function. Transthoracic device closure of VSDs could be considered a safe and feasible technique for surgical repair and transcatheter device closure and had the following advantages: (1) no need for cardiopulmonary bypass; (2) a smaller surgical incision; and (3) quick recovery after surgery. (4) This procedure could be quickly converted to surgical repair if device closure failed [17]. Compared to transcatheter device closure, the greatest advantage of the transthoracic procedure was that it did not require expensive equipment or X-ray exposure. Surgical sternotomy might result in mental and psychological trauma, as well as an unsightly appearance.

At present, most of the studies on the QoL of VSD have focused on children and adolescents [18, 19]. In our previous study, we found that the short-term QoL of adult patients was improved after undergoing transthoracic device closure of VSDs [20]. In this study, the corresponding mid-term QoL of adult patients also showed an improvement trend, and such improvement was not transient. After the intracardiac structural malformations were corrected, the patients gradually adapted to the hemodynamic changes over time. The echocardiography results in this study showed that the size of the patient’s left heart was reduced after surgery. The decrease in the volume load on the left heart improved the patient’s circulation and thus might improve their exercise ability, which also provided a basis for the improvement of their postoperative QoL. In addition, during the survey, some patients said that their self-cognition was no longer significantly different from ordinary people, and they improved in some social activities, such as employment, schooling, or social insurance.

Interactions with patients during our study showed that patients who underwent transthoracic device closure of VSDs had a higher quality of life than patients who underwent surgical repair. Patients who underwent surgical repair tended to be more pessimistic in terms of social interaction and self-evaluation of their physical appearance. This study did not conduct in-depth research about the differences between transthoracic device closure and surgical closure in terms of QOL in patients with small shunts, but such shunt amounts could not affect the patients’ hemodynamics. The purpose of this study was to explore the gap in the quality of life and cardiopulmonary function between adult patients after transthoracic device closure of VSDs and healthy people as a basis for judging the medium-term effectiveness of the treatment. Therefore, healthy adults were selected as the control group in this study.

Some previous studies indicated that compared with healthy people, the health perception of patients with CHD was significantly lower, and the gap in the physical field was more prominent [21, 22]. In our study, the QoL feedback of the patients before treatment was compared with that of the healthy control group, and the patients had lower scores in the four dimensions of “SF”, “VT”, “GH”, and “PF”. Combined with our investigation of the patients’ preoperative status, medical reports of some patients indicated that they had pulmonary hypertension and intracardiac shunts, and these disadvantages in exercise caused by these factors might be associated with their low QoL. After transthoracic device closure, the QoL gap between patients and healthy controls was reduced. When comparing the five-year follow-up, only the “SF” dimension was still significantly different, but there was no significant difference between the two groups in terms of physical and mental component summary scores. These results might indicate that the patient’s QoL was expected to return to the average level to improve symptoms and functions after VSD closure.

In Eckerstrom’s report, patients with VSD were not significantly different from healthy controls at rest, but when the cardiopulmonary system was under pressure, their cardiopulmonary exercise ability was reduced compared with that of healthy controls [23]. In this study, there was no significant difference between the two groups in the pulmonary function test during the 1-year follow-up. However, in the outcome of the cardiopulmonary exercise test, there was still a gap between the patients’ performance under peak exercise and that of healthy controls. The maximal workload and peak oxygen uptake of patients was significantly lower than that of healthy controls. However, under the condition of an absolute anaerobic threshold, there were still differences in oxygen uptake, maximum workload, and heart rate between patients and healthy controls. These results were in line with Heiberg and his colleagues’ findings on exercise capacity in patients with VSD closure [24]. Their results showed the same trend during the five-year follow-up, indicating that the difference in cardiopulmonary function had not been erased over time.

Combined with the results of our survey on QoL and cardiopulmonary exercise tests, we could assume that after receiving transthoracic device closure, the patients’ ability to cope with daily life was improved, and their exercise abilities could be normal. However, due to their impaired aerobic exercise capacity, their capacity for peak exercise still failed to reach a normal level.

During the course of the study, several limitations could be found: (1) most of the patients and participants in this study were from southeast China, so the study’s conclusion might have geographical limitations. (2) Due to the different severities of the disease, it is unknown whether the severity affected the patients’ willingness to participate in the study and whether this bias affected the accuracy of the results. (3) Some of the data were subjective, which could lead to some deviations in the results. (4) The cardiopulmonary exercise test was not routinely performed before VSD closure. (5) this study was a retrospective analysis, which is prone to recall bias and selection bias, and the amount of research data was also limited. And (6) The retrospective design of this study itself made the incremental value of this study shallow. We strive to conduct prospective studies in the future to obtain better incremental value.

## Conclusion

Our study showed that the QoL of adult patients with VSDs was significantly improved after transthoracic device closure, and this improvement was reflected in both the physical and psychological fields. Moreover, such improvement was not transient. However, despite the correction of the pathological defects, a deficiency of cardiopulmonary exercise ability still existed. Therefore, further study and interventions should be considered.

## Data Availability

Data sharing not applicable to this article as no data sets were generated or analyzed during the current study.

## Abbreviations

VSD: Ventricular septal defect
CHD: Congenital heart disease
QoL: Quality of life
PF: Physical functioning
RP: Role physical
BP: Bodily pain
GH: General health
PCS: Physical component summary
VT: Vitality
SF: Social functioning
RE: Role emotional
MH: Mental health
MCS: Mental component summary
